# Effects of Alpha-Lipoic Acid on Oxidative Stress and Kinin Receptor Expression in Obese Zucker Diabetic Fatty Rats

**DOI:** 10.4172/2155-6156.1000556

**Published:** 2015-06-01

**Authors:** Adil El Midaoui, Sébastien Talbot, Karim Lahjouji, Jenny Pena Dias, I. George Fantus, Réjean Couture

**Affiliations:** 1 Department of Molecular and Integrative Physiology, Faculty of Medicine, Université de Montréal, PO Box 6128, Station City-Center, Montréal, Qc, H3C 3J7 Canada; 2 Department of Medicine, Mount Sinai Hospital and University Health Network, Banting and Best Diabetes Center, University of Toronto, Toronto, On, M5G 2C4 Canada

**Keywords:** Alpha-lipoic acid, Diabetes, Kinin receptors, Obesity, Oxidative stress

## Abstract

**Objective:**

To investigate the impact of alpha-lipoic acid on superoxide anion production and NADPH oxidase activity as well as on the expression of kinin B1 and B2 receptors in key organs of obese Zucker Diabetic Fatty rats.

**Methods:**

Superoxide anion production was measured by lucigenin chemiluminescence. Kinin B1 and B2 receptors expression was measured at protein and mRNA levels by western blot and qRT-PCR in key organs of Zucker Diabetic Fatty and Zucker lean control rats treated for a period of 6 weeks with a standard diet or a diet containing the antioxidant α-lipoic acid (1 g/kg).

**Results:**

Superoxide anion production and NADPH oxidase activity were significantly enhanced in aorta and adipose tissue of Zucker Diabetic Fatty rats. Kinin B1 and B2 receptors expression levels were also significantly increased in the liver and the gastrocnemius muscle of Zucker Diabetic Fatty rats. Expression of both receptors was not altered in the pancreas of Zucker Diabetic Fatty rats and was undetectable in white retroperitoneal adipose tissue. Alpha-lipoic acid prevented the rise in NADPH oxidase activity in aorta and epididymal adipose tissue of Zucker Diabetic Fatty rats and the upregulation of kinin B1 receptor in liver and gastrocnemius muscle and that of kinin B2 receptor in the liver. Alpha-lipoic acid treatment was found to prevent the final body weight increase without affecting significantly hyperglycemia, hyperinsulinemia and insulin resistance index in Zucker Diabetic Fatty rats.

**Conclusion:**

Findings support the hypothesis that oxidative stress is implicated in the induction of kinin B1 receptor in Zucker Diabetic Fatty rats. The ability of α-lipoic acid to blunt the body weight gain appears to be mediated in part by preventing NADPH oxidase activity rise in adipose tissue and reversing the hepatic upregulation of kinin B1 receptor in Zucker Diabetic Fatty rats.

## Introduction

Diabetes mellitus (DM) is defined by the World Health Organisation (WHO) as a metabolic disorder of multiple etiology. The global figure of people with diabetes is projected to increase to 333 million in 2025, and 430 million in 2030 [1]. This huge increase in diabetic population is due to aging, adoption of westernised lifestyle, physical inactivity and obesity [2]. Several hypotheses were suggested to explain the enhanced risks associated to diabetes; among these, one of the most plausible is an increase in oxidative stress. Oxidative stress may result from either excessive production of reactive oxygen species (ROS), especially the superoxide anion or from reduced antioxidant reserve. Previous studies have suggested that increased superoxide anion production may be involved in the pathogenesis and complications of diabetes and hypertension [3,4]. Studies have shown that α-lipoic acid (LA), which is a powerful antioxidant, improved the insulin sensitivity in patients with type 2 diabetes [5]. Moreover, the treatment of insulin-resistant Zucker rats with α-lipoic acid was found to increase both oxidative and non-oxidative glucose metabolism and to reduce insulin resistance [6]. In addition, LA was shown to prevent the development of insulin resistance and arterial hypertension as well as to decrease the body weight in chronically glucose-fed rats [7–10]. In obese patients with impaired glucose tolerance and dyslipidaemia, short-term treatment with LA (600 mg intravenously once daily over a period of 2 weeks) improved the insulin sensitivity and the plasma lipid profile [11]. The same treatment was found to decrease the levels of MDA, 8-iso-prostaglandin, TNF-α and IL-6 [11].

Kinins are important vasoactive peptides whose effects are mediated by two G-protein-coupled receptors (R), named kinin B2 receptor (constitutive) and kinin B1 receptor (inducible). They are involved in vascular homeostasis and cardiovascular diseases [12]. The kinin B2 receptor (B2R) is constitutively expressed troughout the body while the kinin B1 receptor (B1R) is virtually absent in healthy tissues, yet this receptor is induced by the cytokine pathway and the oxidative stress via the transcriptional nuclear factor kappa B (NF-κB) [12]. Studies from our laboratory have shown that treatments with LA or N-acetyl-L-cysteine, two potent antioxidants, prevented the upregulation of B1R in central and peripheral tissues in an hypertensive insulin-resistant rat model [8,13]. Moreover, α-lipoic acid therapy or 1-week treatment with SSR240612, a selective B1R antagonist, reduced hyperglycemia, hyperinsulinemia, insulin resistance, hypertension, body and epididymal fat gain, plasma lipids abnormalities and pain neuropathy in chronically glucose-fed rats [8,13–16]. On the other hand, investigations have shown that lack of both kinin B1 and B2 receptors enhanced nephropathy, neuropathy, and bone mineral loss in Akita type 1 diabetic mice [17]. Therefore discrepancies may exist between species and experimental models. The expression and mode of regulation of kinin receptors in Zucker Diabetic Fatty (ZDF) rats, a classical model of type 2 diabetes, have not been investigated to date.

The present study was designed to evaluate the effect of supplemented LA diet (1 g/kg) on: 1- the expression of kinin B1 and B2 receptors at mRNA and protein levels in diabetes target organs (liver, pancreas, gastrocnemius skeletal muscle, white retroperitoneal adipose tissue); 2- superoxide anion production (O_2_^•−^) and NADPH oxidase activity (aorta and epididymal adipose tissue); and 3-plasma levels of glucose and insulin, insulin resistance (HOMA index) and body weight in ZDF rats.

## Materials and Methods

### Animal care and treatments

This study was performed in Obese male ZDF (fa/fa) rats and lean male (ZL, fa/+) rats at 6 weeks of age (Charles River laboratories, St-Constant, Qc, Canada). Four groups of eight rats were divided randomly and treated for 6 weeks as follows: Group 1, ZDF rats were fed standard laboratory chow diet (SD); Group 2, ZDF rats were fed SD supplemented with α-lipoic acid (LA) (1g/kg feed); Group 3, ZL rats were fed SD; Group 4, ZL rats were fed SD supplemented with α-lipoic acid (1g/kg feed). The LA supplemented diet was obtained from Ren’s Feed Supplies Limited (Oakville, On, Canada). All rats were given tap water *ad libitum*. Body weight of each group of rats was measured at the beginning and at the end of the 6-week treatment with SD or LA. However, only data prior to sacrifice were presented to show the impact of LA on final body weight as the initial body weight prior to the 6-week treatment with SD or LA diet was not significantly different between Group 1 and Group 2 (ZDF) and between Group 3 and Group 4 (ZL). The rats were killed by decapitation after light anesthesia with CO_2_. After opening the thorax, blood was withdrawn into a vacutainer tube for plasma biochemistry. All blood samples were drawn early in the morning after fasting overnight (16 h). Organs and tissues (thoracic aorta, liver, pancreas, gastrocnemius skeletal muscle, retroperitoneal and epididymal adipose tissues) were rapidly harvested, frozen in liquid nitrogen and stored at −80°C until subsequent analysis. All research procedures and the care of the animals were in compliance with the guiding principles for animal experimentation as enunciated by the Canadian Council on Animal Care and were approved by the Animal Care Committee of our University.

### Plasma biochemistry

Plasma glucose concentrations were measured with a glucometer (Elite, Bayer Inc). Insulin levels were determined by radioimmunoassay (rat insulin RIA kit, Linco Research, St. Charles, USA) using 100 μL of plasma. To estimate the degree of insulin resistance, we have used the Homeostasis Model Assessment (HOMA) index as calculated by the following formula: [insulin (in μU/mL) × glucose (in mmol/L)]/22.5 [18].

### Oxidative stress measurement

Production of superoxide anion (O_2_^•−^) was measured in thoracic aorta and epididymal fat tissue using the lucigenin-enhanced chemiluminescence method as described previously [19,20]. Briefly, for each animal, small slices from aorta and epididymal fat tissue were preincubated in Krebs–HEPES buffer (saturated with 95% O_2_ and 5% CO_2_, at room temperature for 30 min) and then transferred to glass scintillation vials containing 5 μmol/L lucigenin for the determination of basal O_2_^•−^ levels. Lucigenin counts were expressed as counts per minute (cpm) per milligram of mass of tissue. Moreover, the activation of NADPH oxidase in the samples was assessed by adding 0.1 mmol/L NADPH to the vials before counting. Basal superoxide-induced luminescence was subtracted from the luminescence value induced by NADPH.

### Western blot

Tissue protein quantification was performed with a bichinconic acid (BCA) kit (Thermo Scientific) using bovine serum albumin (BSA) as standard. Samples were run by electrophoresis on a 10% polyacrylamide gel and transferred onto nitrocellulose membranes. The efficiency of the overall procedure was monitored by Ponceau red staining. The membranes were blocked with a commercial blocking buffer (Thermo Scientific) in Phosphate Buffer Saline S-Tween 20, 0.1% (PBS-T). Membranes were then probed with our in house rabbit anti-B1R [21–23] or mouse anti-B2R (Cat no 610451, BD Transduction Laboratories, Lexington. USA) antibody diluted 1:1000. The secondary antibodies for B1R and B2R identification were an Horseradish Peroxidase (HRP)-linked goat anti-rabbit and an HRP-linked goat anti-mouse (Santa Cruz Biotech, CA, USA) used at a 1:25000 dilution, respectively. Antibodies incubation was performed in the commercial blocking buffer. Membranes were rinsed adequately between every step with PBS-T and revealed using an Enhanced Chemiluminescence Detection System (Super-Signal^®^, Thermo Scientific, Canada). Dynein was used as standard protein and revealed with mouse anti-dynein monoclonal antibody (Santa Cruz Biotech, CA, USA) diluted 1:25000. HRP-linked goat anti-mouse (Santa Cruz Biotech, CA, USA) was used as secondary antibody at a 1:25000 dilution. A quantitative analysis of the detected protein was performed by densitometry using an MCID^™^ image analysis system (Imaging Research, St. Catharines, On, Canada).

### Total RNA extraction and SYBR green-based quantitative RT-PCR

The protocol for mRNA extraction, cDNA generation, SYBR green-based quantitative RT-PCR and quantification was described elsewhere [14,15]. Briefly, the PCR conditions were as follows: 95°C for 15 min, followed by 46 cycles at 94°C for 15 s, 60°C for 30s and 72°C for 30s. The Vector NTI-designed qPCR primers pairs are shown in Table 1. For standardization and quantification, rat 18S was amplified simultaneously. Relative gene expression values were expressed as fold change and were obtained by comparing ZDF to ZL rats.

### Statistical analysis

Data were expressed as the mean ± SEM of values obtained from *n* rats. Statistical significance was determined with a one-way analysis of variance (ANOVA) followed by the *post-hoc* Bonferonni or Tukey-test for multiple comparisons and two-tailed unpaired student’s *t*-test for single comparison. Only probability (P) values less than 0.05 were considered to be statistically significant.

## Results

### Effects of α-lipoic acid (LA) on body weight and metabolic parameters

As expected, the final body weight was significantly increased (P<0.01) in ZDF rats in comparison with ZL rats (Table 2). The supplementation of the diet with LA had no effect on this parameter in ZL rats, whereas it prevented the increase in final body weight in ZDF rats. As shown in Table 2, ZDF rats showed marked and significant increases (P<0.01) in plasma glucose and insulin levels as well as insulin resistance (HOMA index) in comparison with ZL rats. The treatment with LA did not affect significantly plasma glucose and insulin levels and insulin resistance in ZDF rats. Also, LA had no significant effect on these parameters in ZL rats.

### Effects of α-lipoic acid on oxidative stress parameters

**Figure 1:**
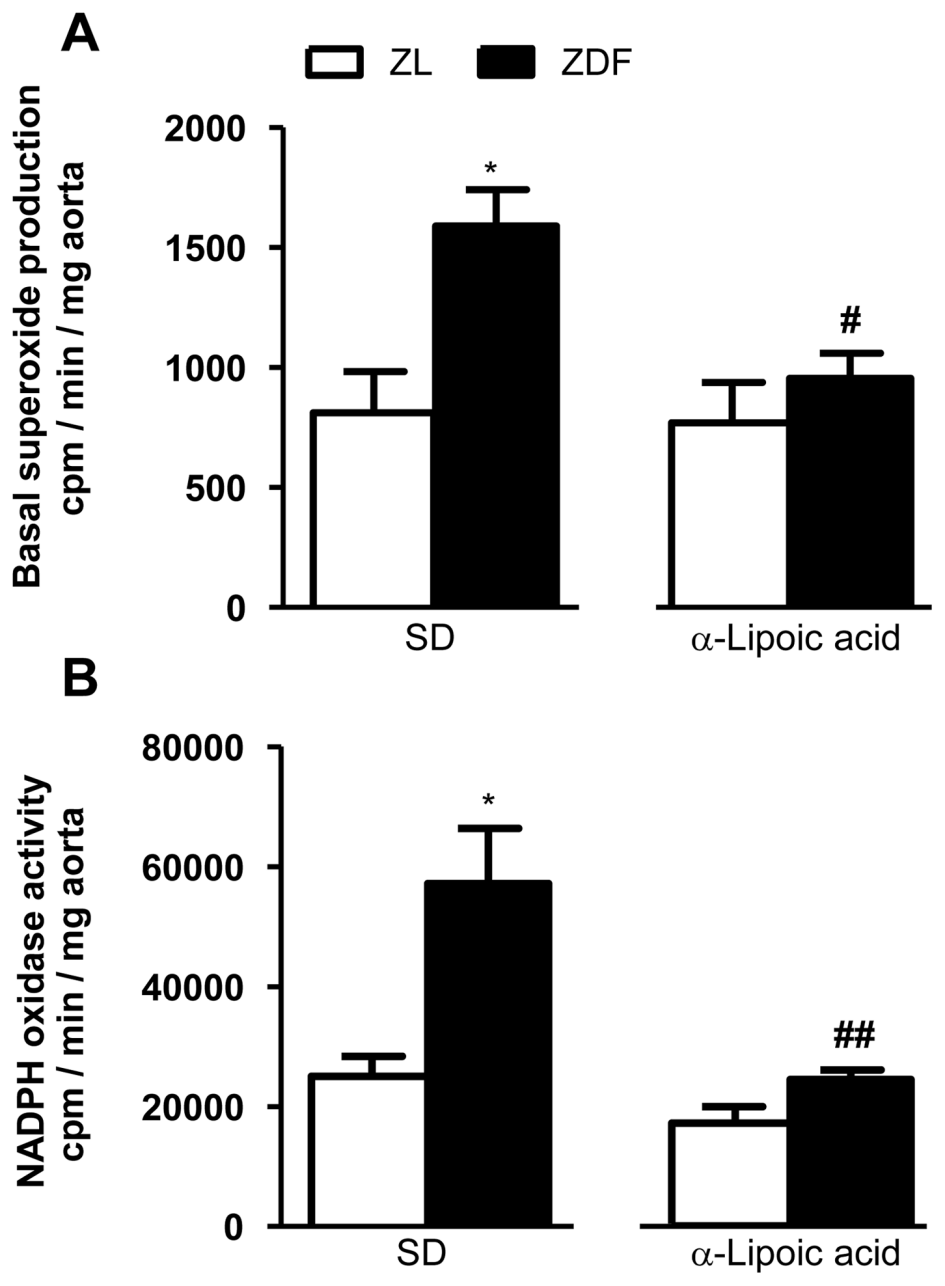
Effect of 6-week treatment with α-lipoic acid or standard diet (SD) on superoxide anion production (A) and NADPH oxidase activity (B) in aorta of 12 weeks Zucker diabetic fatty (ZDF) rats and age matched Zucker lean (ZL) rats. ^*^P<0.05 vs ZL rats; **^#^**P<0.05 and **^##^**P<0.01 vs ZDF rats.

**Figure 2:**
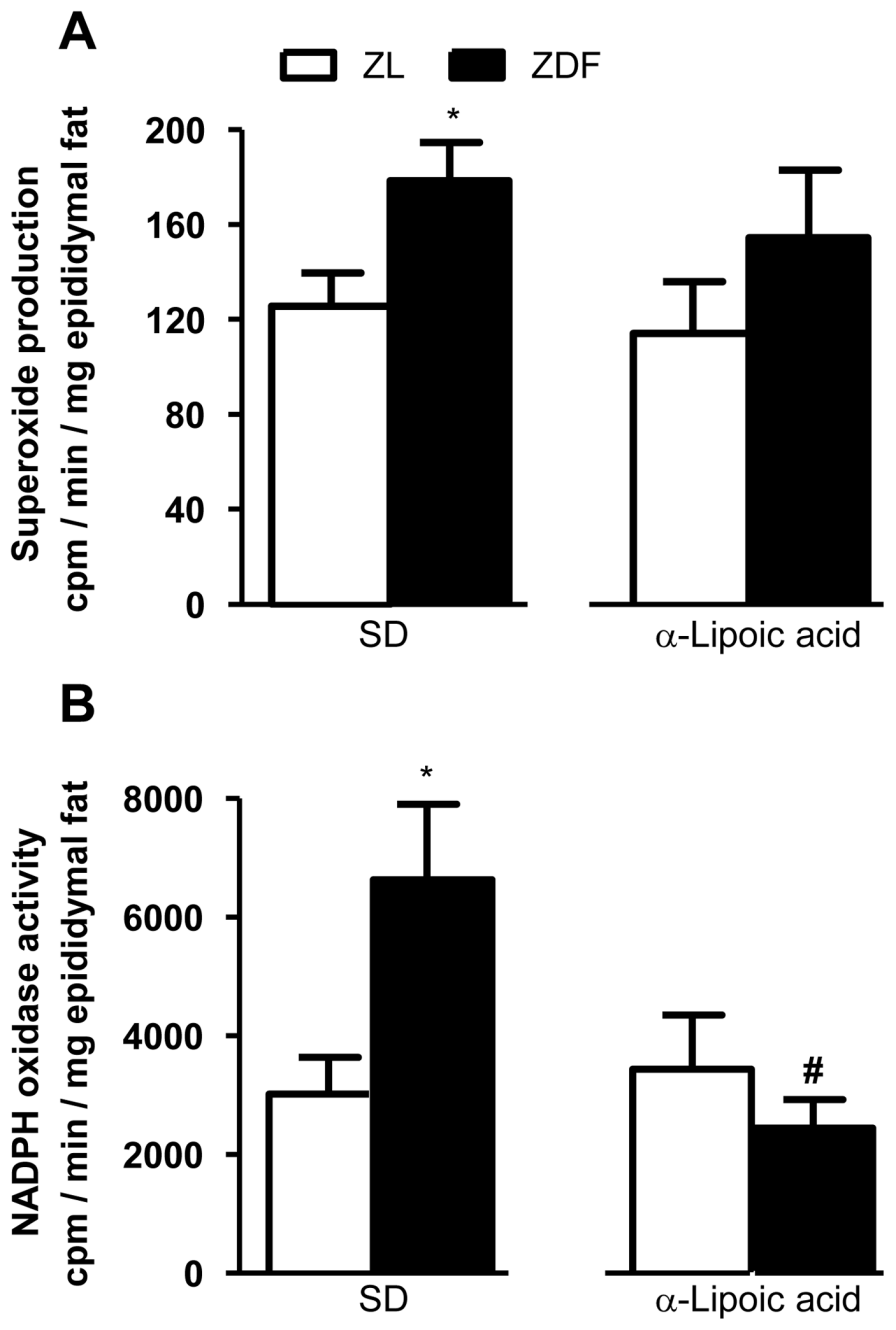
Effect of 6-week treatment with α-lipoic acid or standard diet (SD) on superoxide anion production (A) and NADPH oxidase activity (B) in epididymal fat tissue of 12 weeks Zucker diabetic fatty (ZDF) rats and age-matched Zucker lean (ZL) rats. ^*^P<0.05 vs ZL rats; **^#^**P<0.05 vs ZDF rats.

As shown in Figure 1A and 2A, basal superoxide anion production was significantly (p <0.05) increased in aorta and epididymal fat tissues in ZDF rats in comparison with ZL rats. Six weeks of treatment with LA blocked significantly the rise in superoxide anion production in aorta and the difference between ZL and ZDF was no longer significant in the epididymal fat**.** As shown in Figures 1B and 2B, NADPH oxidase activity was significantly (p < 0.05) increased in aorta and epididymal fat tissues of ZDF rats in comparison with ZL rats. Six weeks of treatment with LA abolished the rise in NADPH oxidase activity in both aorta and epididymal fat tissue in ZDF rats.

### Effect of α-lipoic acid on kinin receptors expression

**Figure 3:**
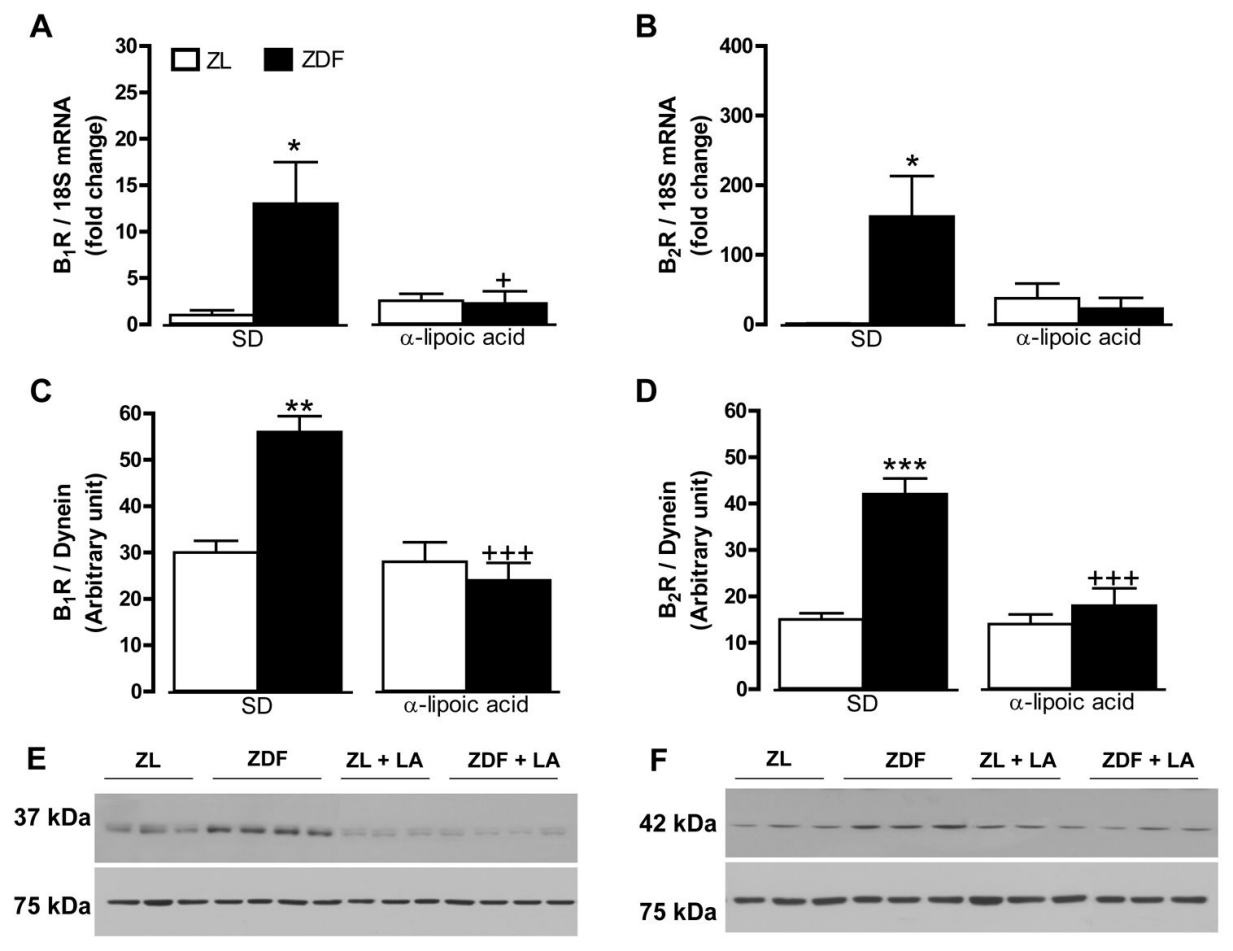
Effect of 6-week treatment with α-lipoic acid (LA) or standard diet (SD) on the expression of kinin B1R (A, C, E) and B2R (B, D, F) in the liver of 12 weeks Zucker diabetic fatty (ZDF) rats and age-matched Zucker lean (ZL) rats. Shown are mRNA (A, B) and protein (C–F) expression levels of B1R (37 kDa) and B2R (42 kDa). Dynein (75 kDa) and 18S mRNA were used as standards. Data are the mean ± S.E.M of 3–8 rats/group. Statistical comparison with ZL (*) and ZD (+) in SD is indicated by *,+ P<0.05; ** P <0.01; ***,+++ P<0.001.

**Figure 4:**
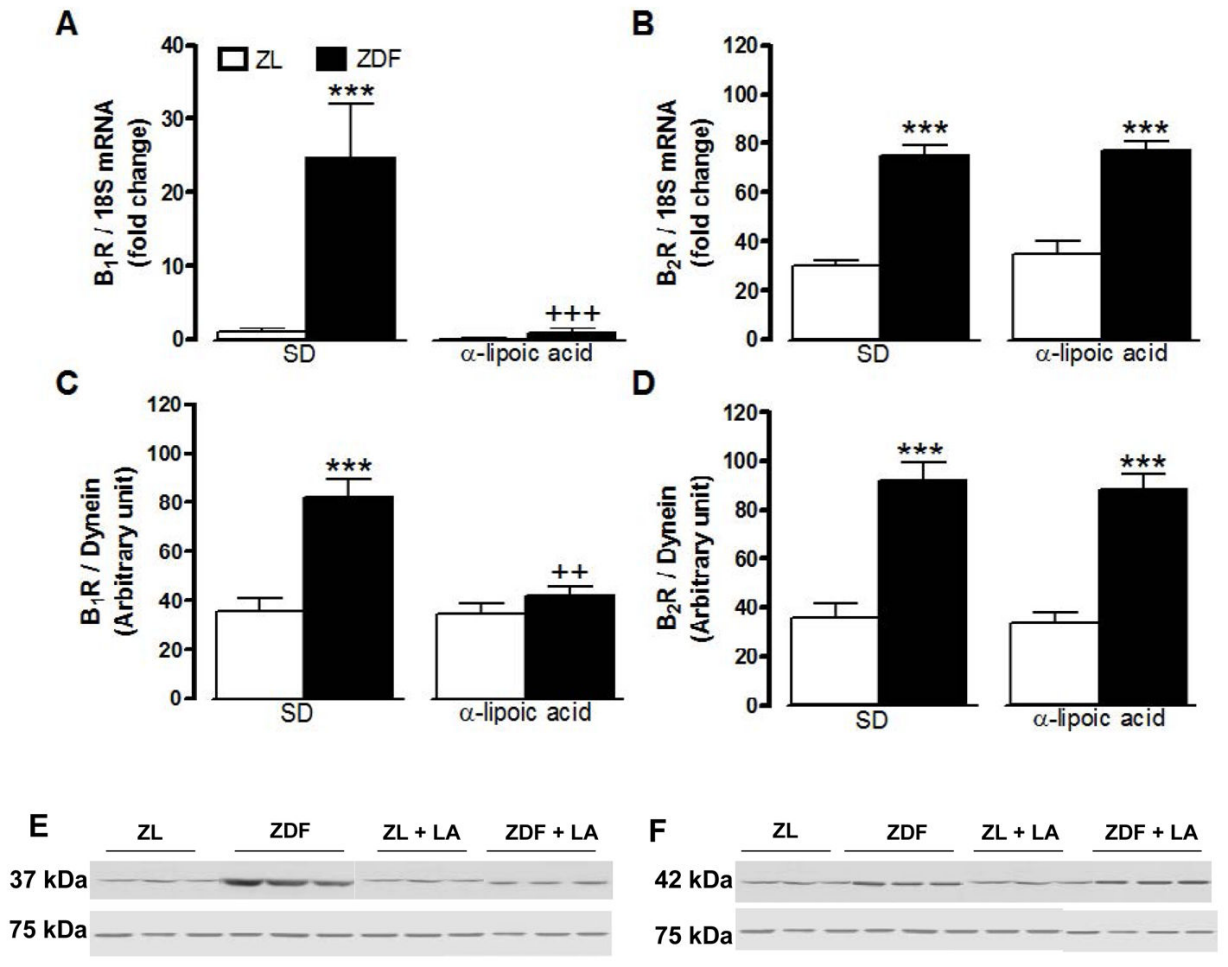
Effect of 6-week treatment with α-lipoic acid (LA) or standard diet (SD) on the expression of kinin B1R (A, C, E) and B2R (B, D, F) in the gastrocnemius skeletal muscle of 12 weeks Zucker diabetic fatty (ZDF) rats and age-matched Zucker lean (ZL) rats. Shown are mRNA (A, B) and protein (C–F) expression levels of B1R (37 kDa) and B2R (42 kDa). Dynein (75 kDa) and 18S mRNA were used as standards. Data are the mean ± S.E.M of 3–8 rats/group. Statistical comparison with ZL (*) and ZD (+) in SD is indicated by ++ P<0.01; ***,+++ P<0.001.

**Figure 5:**
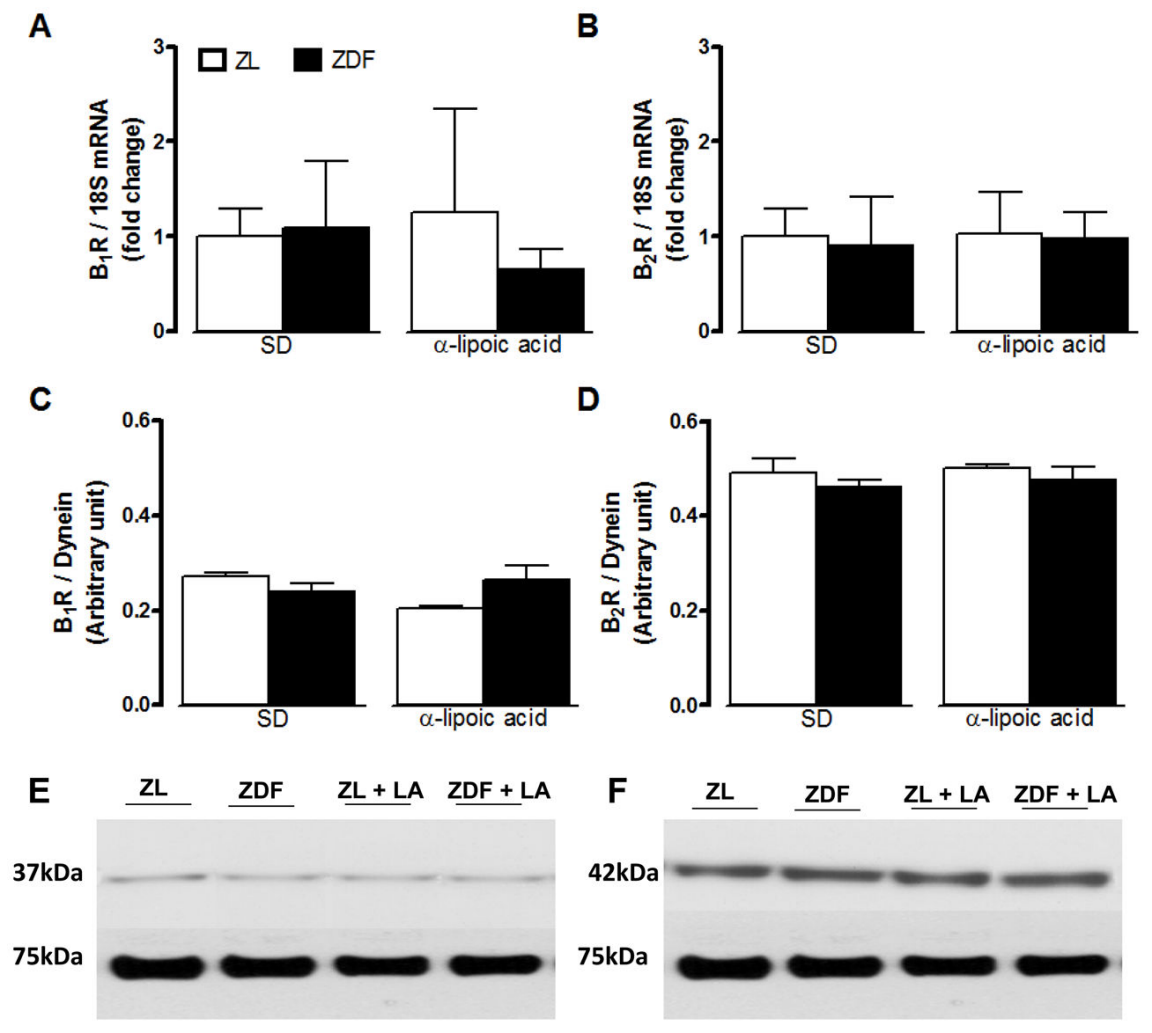
Effect of 6-week treatment with α-lipoic acid (LA) or standard diet (SD) on the expression of kinin B1R (A, C, E) and B2R (B, D, F) in the pancreas of 12 weeks Zucker diabetic fatty (ZDF) rats and age-matched Zucker lean (ZL) rats. Shown are mRNA (A, B) and protein (C–F) expression levels of B1R (37 kDa) and B2R (42 kDa). Dynein (75 kDa) and 18S mRNA were used as standards. Data are the mean ± S.E.M of 3–8 rats/group.

**Figure 6:**
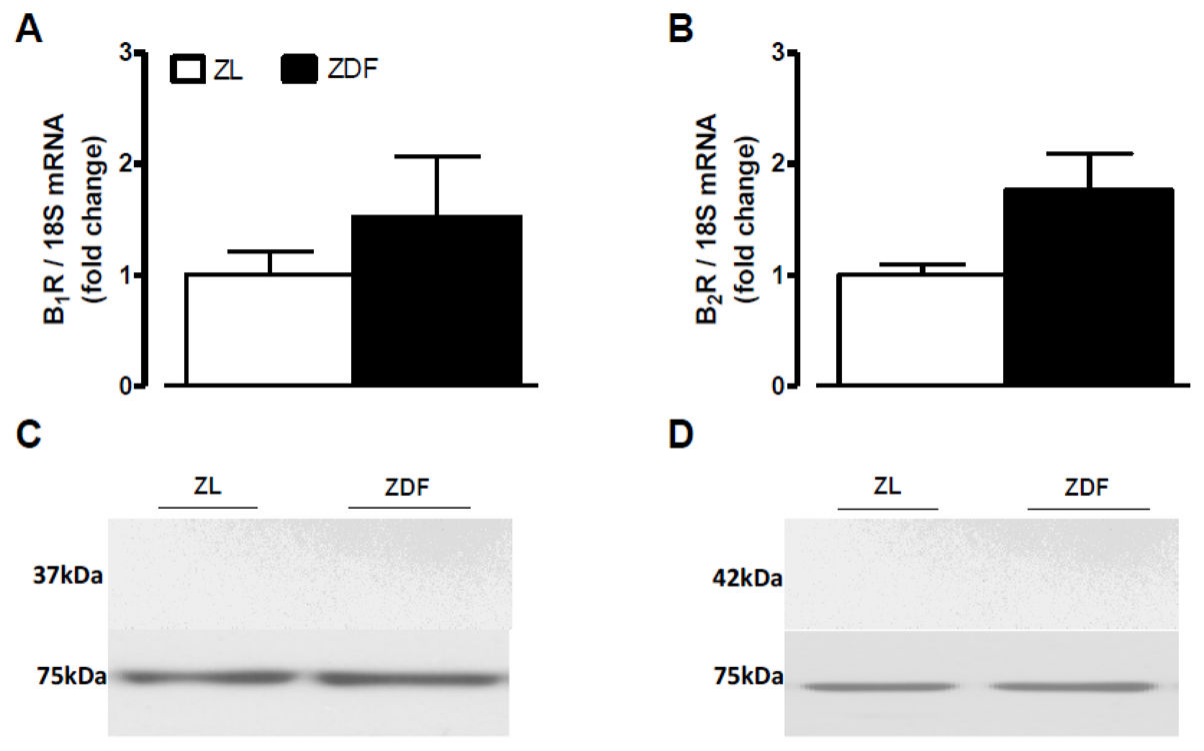
Expression levels of kinin B1R (A, C) and B2R (B, D) in the white adipose tissue of 12 weeks Zucker diabetic fatty (ZDF) rats and age-matched Zucker lean (ZL) rats. Shown are mRNA (A, B) and protein (C, D) expression levels of B1R (37 kDa) and B2R (42 kDa). Dynein (75 kDa) and 18S mRNA were used as standards. Data are the mean ± S.E.M of 3–8 rats/group.

B1R and B2R mRNA and protein expression levels were significantly increased in the liver (Figure 3) and the gastrocnemius muscle (Figure 4) of ZDF rats in comparison with ZL rats. A 6-week treatment with LA prevented the raised in B1R at mRNA and protein levels in the liver and gastrocnemius of ZDF rats while it had no effect on basal B1R expression in ZL rats (Figures 3 and 4). Protein and mRNA levels of B2R were reversed by LA in the liver but not in the gastrocnemius of ZDF rats (Figures 3 and 4). As shown in Figure 5, protein and mRNA expression levels of B1R and B2R in the pancreas were not significantly different in ZDF in comparison to ZL rats. Moreover, LA treatment had no effect on these parameters in those animals (Figure 5). Both B1R and B2R failed to be detected at mRNA and protein levels in the white adipose tissue of ZL and ZDF rats (Figure 6).

## Discussion

The major findings of the present study are the following: 1- B1R and B2R expression at mRNA and protein levels were increased in the liver and the gastrocnemius muscle of ZDF rats, two key targets for fat and glucose metabolism; 2- NADPH oxidase activity and superoxide anion production were increased in aorta and adipose tissue of ZDF rats; 3- supplementation of LA in the diet prevented the rise in B1R at the mRNA and protein expression levels in liver and gastrocnemius muscle in ZDF rats; 4- LA reversed the increase in NADPH oxidase activity and superoxide anion in aorta and adipose tissue in ZDF rats; 5- the suppression of the NADPH oxidase activity and B1R expression with LA was accompanied by a dramatic reduction of body weight in ZDF rats, yet LA treatment failed to affect hyperglycemia, hyperinsulinemia and insulin resistance in ZDF, a classical rat model of type 2 diabetes.

We have previously reported in a rat model of insulin resistance induced by chronic glucose feeding [8,13] and in hypertension induced by chronic infusion of angiotensin II [24] that the oxidative stress contributes to the induction and up-regulation of B1R in various tissues. The present study demonstrated that the treatment with LA prevented the increased expression of B1R along with the enhanced basal production of superoxide anion and NADPH oxidase activity in ZDF, supporting that the oxidative stress is also implicated in the induction and up-regulation of B1R in ZDF rats. This is in agreement with previous studies, which have demonstrated that the superoxide anion production is increased in the aorta and mesenteric arteries from ZDF rats [25,26].

While B1R and B2R were found to be absent in adipose tissue of ZDF rats as reported in insulin-resistant rats [16], B1R mRNA expression was increased and that of B2R decreased in adipose tissue of obese mice lacking leptin gene (ob/ob) [27]. These findings highlight species discrepancies regarding the presence of kinin receptors in adipose tissues. In addition, B1R mRNA levels were found to be increased in aorta and liver but not in skeletal muscle in ob/ob mice while B2R mRNA was not modified in those tissues [27]. Therefore, it appears that kinin receptors are regulated differently in ob/ob mice and ZDF rats even if both have defect in leptin function.

It is well known that the ZDF rat which represents a genetic model of metabolic syndrome suffers from obesity, type 2 diabetes, hypertension and dyslipidemia. Investigations have shown that treatment with resveratrol, a potent antioxidant reduced dyslipidemia and TNF-α in visceral adipose tissue of obese Zucker rats [28]. Importantly, we have shown in the present study that LA prevented the rise in the final body weight in ZDF rats. This is in agreement with a recent study which had demonstrated that LA induced prominent gene expression changes in liver in support of significant improvement of whole-body lipid status [29]. In addition, the present study showed that LA treatment prevented the increase in the NADPH oxidase activity in epididymal fat tissue as well as the rise in the liver B1R expression at mRNA and protein levels. This is in agreement with previous studies which have shown that lipoic acid attenuated the rise in vascular superoxide production in ZDF rats [26]. Thus, the present results suggest that oxidative stress through an overexpression of B1R is implicated in the elevation of body weight in ZDF rats. Our data support previous claims showing that B1R knockout mice or chronic blockade of B1R with SSR240612 decreases fat mass, increase energy expenditure and leptin sensitivity in mice treated with high-fat diet [30]. Similarly, 1-week treatment with the B1R antagonist SSR240612 reduced whole body and epididymal fat mass in high glucose feeding rats [16], supporting a role for B1R in obesity associated with type 2 diabetes.

In our study, B2R expression was also increased in the liver of ZDF rats by a mechanism sensitive to LA treatment. However, evidence suggests rather a protective role for B2R in insulin resistance and diabetes. The B2R is known to ameliorate insulin resistance by increasing glucose uptake and supply, and by inducing glucose transporter-4 translocation either directly or through phosphorylation of insulin receptor [31]. Resistance to insulin occurs in B2R knockout mice, which is not compensated by the B1R [32]. B2R also provides cardio- and nephro-protection in ZDF rats [33,34]. Obese mice (ob/ob) lacking the B2R have similar body weight or fat accumulation, but showed increased fasting glycemia and impaired glucose tolerance when compared with ob/ob control mice, indicating insulin resistance and impaired glucose homeostasis [35].

In the present study, LA treatment was found to have no effect on hyperglycemia, hyperinsulinemia as well as the insulin resistance index even though it prevented completely the rise in superoxide anion production and NADPH oxidase activity in aorta and adipose tissue as well as the upregulation of B1R expression in liver and gastrocnemius muscle of ZDF rats. This is in agreement with a recent study [36], which has demonstrated that LA treatment did not decrease the rise in blood glucose levels and insulin resistance while it decreased the elevation of oxidative stress markers as reflected by the reduction in the protein carbonyls in skeletal muscle as well as in the urine conjugated dienes in obese female Zucker rats. Thus, the present study suggests that other mechanisms than B1R and oxidative stress are implicated in alteration of glucose metabolism in ZDF rats.

## Conclusions

The present study demonstrated that supplementation diet with LA prevented the upregulation of B1R in the liver and the gastrocnemius muscle indicating that oxidative stress is implicated in the induction of B1R in ZDF rats. In addition, treatment with LA prevented the increase in body weight but not hyperglycemia, hyperinsulinemia and insulin resistance in ZDF rats. The anti-obesity effect of LA seems to be associated with its anti-oxidative properties and suppression of B1R induction. Conversely, B1R and the oxidative stress do not appear to contribute to insulin resistance or glucose metabolic alteration in ZDF rats.

## Figures and Tables

**Table 1:** qPCR primer pairs sequences.

	Sequences			Position			Gen Bank Accession number
18S Forward	5′	TCA ACT TTC GAT GGT AGT CGC CGT	3′	363	–	386	X01117
18S Reverse	5′	TCC TTG GAT GTG GTA GCC GTT TCT	3′	470	–	447	
B1 receptor Forward	5′	GCA GCG CTT AAC CAT AGC GGA AAT	3′	367	–	391	NM_030851
B1 receptor Reverse	5′	CCA GTT GAA ACG GTT CCC GAT GTT	3′	478	–	454	
B2 receptor Forward	5′	AGG TGC TGA GGA ACA ACG AGA TGA	3′	882	–	905	NM_173100
B2 receptor Reverse	5′	TCC AGG AAG GTG CTG ATC TGG AAA	3′	990	–	967	

**Table 2:** Effect of 6-week treatment with α-lipoic acid (LA) on basal metabolic parameters in 12 weeks old Obese Zucker diabetic fatty (ZDF) rats and age-matched Zucker lean (ZL) rats.

	ZL			ZL + LA			ZDF			ZDF + LA		
Final body weight (g)	312	±	6	305	±	6	365	±	9 **	326	±	5 ++
Plasma glucose (mmol/L)	5.8	±	0.4	5.3	±	0.3	16.8	±	1.8**	12.9	±	1.7**
Plasma insulin (pmol/L)	471.5	±	36.5	520.6	±	130.2	2098.9	±	313.5**	2087.0	±	253.9**
HOMA index	18.0	±	1.9	17.5	±	4.3	271.9	±	30.9**	182.4	±	50.9**

Data are means ± SEM obtained from 8 rats in each group.

** P < 0.01 vs ZL rats,

++ P < 0.01 vs ZDF rats.

Intra-assay coefficient of variance for glucose and insulin in each group are: ZL (19.51, 21.90), ZL + LA (16.01, 70.74), ZDF (30.30, 42.52) and ZDF + LA (37.27, 34.29), respectively.
